# Filling gaps with construction of a genetic linkage map in tetraploid roses

**DOI:** 10.3389/fpls.2014.00796

**Published:** 2015-01-13

**Authors:** Chao Yu, Le Luo, Huitang Pan, Xuelian Guo, Huihua Wan, Qixiang Zhang

**Affiliations:** Beijing Key Laboratory of Ornamental Plants Germplasm Innovation and Molecular Breeding, National Engineering Research Center for Floriculture, Beijing Laboratory of Urban and Rural Ecological Environment and College of Landscape Architecture, Beijing Forestry UniversityBeijing, China

**Keywords:** comparison, EST-SSR, genetic linkage, rose, tetraploid

## Abstract

Rose (*Rosa* sp.) is one of the most economically important ornamental crops worldwide. The present work contains a genetic linkage map for tetraploid roses that was constructed from an F_1_ segregation population using AFLPs and SSRs on 189 individuals. The preliminary ‘Yunzheng Xiawei’ and ‘Sun City’ maps consisted of 298 and 255 markers arranged into 26 and 32 linkage groups, respectively. The recombined parental maps covered 737 and 752 cM of the genome, respectively. The integrated linkage map was composed of 295 polymorphic markers that spanned 874 cM, and it had a mean intermarker distance of 2.9 cM. In addition, a set of newly developed EST-SSRs that are distributed evenly throughout the mapping population were released. The work identified 67 anchoring points that came from 43 common SSRs. The results that were produced from a large number of individuals (189) and polymorphic SSRs (242) will enhance the ability to construct higher density consensus maps with the available diploid level rose maps, and they will definitely serve as a tool for accurate QTL detection and marker assisted selection.

## Introduction

Rose (*Rosa* sp.), which belongs to the family Rosaceae, is one of the most economically important ornamental crops worldwide. The rose has great cultural significance and many desirable ornamental traits, which mean it has been used widely as a symbol and a woody ornamental garden plant as well as a cut flower. In addition, due to *Rosa* sp. having a relatively small genome, great morphological diversity, a short life cycle and several unique ornamental traits, such as a nuance-rich spectrum of flower colors, recurrent blooming and abundant flower fullness, they are regarded as ideal ornamental model species for scientific research (Debener and Linde, 2009).

Recently, the genome of *Rosa chinensis* ‘Old Blush’ has been sequenced, and databases of expressed sequenced tags (ESTs) and digital expressions (RNA Seq) from various developmental stages of flower tissues have been obtained (Dubois et al., 2012; Kim et al., 2012; Pei et al., 2013b; Yan et al., 2014). The constant manipulations that are being performed on diploid rose cultivars show the need to continue developing our understanding of this species with the tools that have become available in the new omics era, which has been done on other model species, such as *Arabidopsis thaliana*, tobacco or maize.

Modern rose cultivars are generally autotetraploids that have a high level of heterozygosity throughout their cultivation history, which is in contrast to the diploid to decaploid wild *Rosa* species (Jian et al., 2013; Yu et al., 2014). Current rose breeding work is mainly performed at the tetraploid level, which means the maps obtained in wild diploid *Rosa wichurana* (Crespel et al., 2002) are not suitable for the QTL detection of ornamental traits. Therefore, it is important that the genetic and molecular mechanisms in modern rose cultivars at the tetraploid level are understood, and this will build on the important framework identified at the diploid level (Gar et al., 2011).

The construction of genetic linkage maps has already been initiated in some well-known ornamental plants and has been rapidly developed among several new flower crops in recent years. As for roses, at both the diploid and tetraploid levels, genetic maps have already been produced. Work on the first rose map started in 1999, and it continues to this day, having already produced 10 maps (Debener and Mattiesch, 1999; Debener et al., 2001; Rajapakse et al., 2001; Crespel et al., 2002; Dugo et al., 2005; Yan et al., 2005a; Linde et al., 2006; Zhang et al., 2006; Hibrand-Saint Oyant et al., 2008; Gar et al., 2011; Spiller et al., 2011; Hosseini Moghaddam et al., 2012; Koning-Boucoiran et al., 2012). The number of mapping populations, the type of molecular marker, the map length covered, the map density and the QTLs related to major rose traits have increased gradually. However, because more than half of the mapping populations are still of small size (<100), inaccurate marker order may have been identified due to inversions and low marker distance (Jairin et al., 2013). Thus, attempts to co-localize a candidate gene and a specific locus have not been successful (Bendahmane et al., 2013). What is more, most of the currently available markers are useful only when incorporating the gene from the specific germplasm source in which the marker was discovered (Debener and Byrne, 2014). This may be attributed to two causes: first, the range of applications is related to the inheritance pattern of the markers. Therefore, the use of co-dominant SSRs that are extremely reproducible is considered to be the best option for producing genetic linkage maps or integrating related maps (Lu et al., 2012; Sun et al., 2013). Second, when there is significant divergence between the parent plants, this may inhibit the chromosomes' exchange and recombination, which could result in the linkage maps being less credible and their scope being reduced (Lu et al., 2012). Nevertheless, more advanced genomic tools combined with more mapping populations with larger sizes and next generation molecular markers will definitely lead to higher-resolution maps that have a sufficient amount of user-friendly DNA markers: this will mean that marker assisted selection will become much easier (Li et al., 2010; Guo et al., 2014).

We initiated a rose breeding and genomics project about seven years ago that was aimed at identifying the desired traits and genes from germplasm native to China, including wild *Rosa* species and old garden rose cultivars. After a 4 year field investigation, resource evaluation and cross breeding work, we decided to construct a genetic linkage map at the tetraploid level with a large segregating population. This had three objectives: (1) to provide an important framework at the tetraploid level to search for QTLs related to ornamental traits; (2) for map-based marker assisted selection use; and (3) to provide essential data for the forthcoming genome assembly and arrangement.

This work used a large number of individuals (189) to develop a tetraploid level genetic linkage map from AFLPs and SSRs. In addition, a comparison with the consensus diploid map was conducted to find anchoring points for a higher-density genetic linkage map in roses.

## Materials and methods

### Mapping population

**Figure 1:**
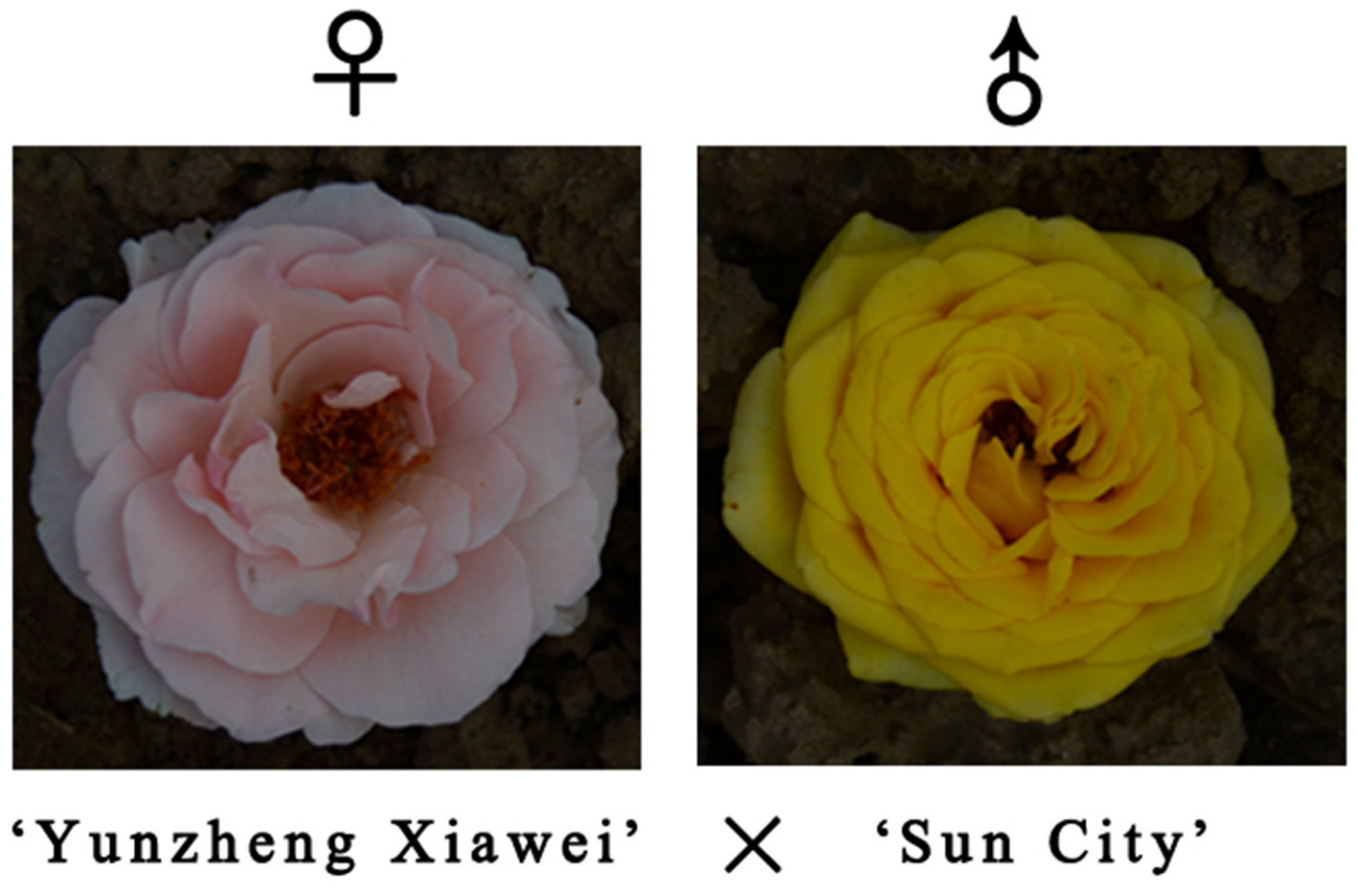
**Flowers of the parental genotypes of the mapping population**. Chinese old garden rose cultivar *Rosa chinensis* ‘Yunzheng Xiawei’ (left) and the modern rose cultivar ‘Sun City’ (right).

A cross between the Chinese old garden rose cultivar *R. chinensis* ‘Yunzheng Xiawei’ and the modern rose cultivar ‘Sun City’ was used for raising the tetraploid mapping population (Figure 1). Despite not being as well-known as ‘Old Blush’ or ‘Viridiflora,’ the female parent ‘Yunzheng Xiawei’ is an old Chinese garden rose that can be useful as a tetraploid cultivar that has white-pink flowers and moderate fragrance. The male parent, ‘Sun City,’ has a star-shaped, deep yellow flower that has attracted large-scale popularity in the market (Cairns, 2000). The F1 mapping population was formed in 2012 by randomly selecting 189 individuals from a total population of 333 plants. Due to the woody plants being extremely heterozygous, a pseudo testcross mapping strategy was used (Grattapaglia and Sederoff, 1994).

The cross breeding was performed in Kunming, southwest China from 2008 to 2010. The progenies were cultivated and grown in the Xiao Tangshan horticultural fields, affiliated to Beijing Forestry University, Beijing, China. Total DNA was extracted from fresh young leaves with a plant genomic DNA extraction kit (TIANGEN) following the manufacturer's instructions. The quality of the extracted DNA was verified by 1% agarose gel electrophoresis. The DNA samples were stored at −20°C.

### AFLP protocol

The primer combinations used in the AFLP analysis are shown in Table 1. The method of Vos et al. (1995), with some modifications, was used with *Eco*RI and *Mse* I. In this method, 200 ng of DNA was digested in a final volume of 15 μl at 37°C for 12 h with 1.6 U of *Eco*RI, 0.9 U of *Mse* I, 2.0 μl 10 × T4 DNA ligase Buffer, 0.33 μmol/L *Eco*RI adaptors, 3.3 μmol/L *Mse* I adaptors and 0.6 U T4 DNA ligase. A pre-amplification reaction was performed with primers complementary to each adapter that had an additional selective nucleotide, specifically *Eco*RI adaptor +C and *Mse*I adaptor +A. The pre-amplification reaction was performed in a total volume of 20 μl with 2 μl template DNA, 0.4 mmol/L *Eco*RI and *Mse* I pre-amplification primer, 2 μl 10 × PCR Buffer, 1 U Hs *Tag* DNA polymerase (Microread, Beijing, China) and 0.9 mmol/L dNTP. The PCR amplification consisted of 95°C for 5 min; 30 cycles of 95°C for 30 s, 56°C for 30 s, and 72°C for 1 min; and a final extension step at 72°C for 5 min. The pre-amplification products were diluted 20 times with TE buffer, and 4 μl was used for selective amplification, in which the cycle profile was as follows: 95°C for 5 min; 30 cycles of 95°C for 30 s, 56°C for 30 s, and 72°C for 2 min; and a final extension step at 72°C for 5 min. The fragment patterns were firstly electrophoresed on a 6% denatured polyacrylamide gel and then visualized using silver staining. Then primers with clear and polymorphic fragments were labeled with a fluorophore (HEX or FAM) for selective amplification without a test for reproducibility.

**Table 1:** **Total number of AFLPs and polymorphic markers generated with 10 different primer combinations**.

**Primer code**	**Primer combinations**	**Total fragments**	**Polymorphic markers**		**Polymorphism (%)**
			**Y**	**T**	
E1M1	E-AAC + M-CAA	23	10	8	78.26
E2M1	E-ACA + M-CAA	39	9	10	48.72
E3M1	E-AGG + M-CAA	33	10	5	45.45
E4M2	E-ATC + M-CCA	37	11	7	48.65
E1M3	E-AAC + M-CAG	91	18	20	41.76
E2M3	E-ACA + M-CAG	28	6	6	42.86
E3M7	E-AGG + M-CAC	29	6	9	51.72
E4M4	E-ATC + M-CGA	50	8	10	36.00
E6M3	E-AAG + M-CAG	44	8	13	47.73
E5M7	E-ACT + M-CAC	65	12	20	49.23
Total	–	439	98	108	46.92
Average	–	43.9	20.6		–

### SSR protocol

A total of 697 SSRs were analyzed, of which 441 EST-SSRs were developed from the public EST database and 256 pairs were identified from previous studies (Esselink et al., 2003; Suss and Schultze, 2003; Zhang et al., 2006; Hibrand-Saint Oyant et al., 2008). The newly developed 441 pairs of EST-SSRs were given numerical identifiers from 301 to 716 (416 pairs), while the other 25 pairs began with ‘RH’ according to the EST names.

All the SSRs were screened for polymorphisms among six randomly chosen segregating individuals and the two parental samples. The PCR amplification reactions were conducted in a total volume of 20 μl containing 100 ng of DNA, 10 μl 2 × Taq PCR Master Mix (Biomiga), 0.5 μmol/L each of the forward and reverse primers and ddH_2_O to the total volume. The following thermocycling conditions were used in the PCR: an initial denaturation at 94°C for 3 min; 30 cycles of 94°C for 30 s, primer-specific temperature for 30 s and 72°C for 1 min; and a final extension step at 72°C for 10 min. The product was firstly run on a 1% agarose gel, if fragments with the expected size were present, then the product was electrophoresed on a 6% denatured polyacrylamide gel and finally silver stained to visualize the fragments. The SSRs that generated reproducible polymorphisms were then used with all the 191 samples (189 segregating individuals and two parents). The subsequent genotyping work was performed using a three-primer strategy as detailed in the protocol of Sun et al. (2013).

The AFLP and SSR products (1 μ) were then analyzed on an ABI3730 fluorescent analyzer with 0.5 μl Rox 500 HD (Microread) size standard and 8.5 μl Hi-Di formamide. The data were analyzed using GeneMapper (version 4.0).

### Linkage analysis and map construction

Alleles were read independently and scored as ‘1’ or ‘0’ for presence or absence, respectively. Each marker was tested for the expected for simplex (single dose) and duplex (double dose) segregation ratios under the possible inheritance patterns. For both uni-parental and bi-parental markers, only the simplex allele was included in the mapping and construction of the genetic maps. A Chi-square (χ^2^) test of goodness-of-fit was performed on the segregation data at the 5% significant level. The segregation of markers that did not fit the ratio was treated as distorted. Markers that segregated in a Mendelian fashion or deviated only slightly from it were used for map construction that was carried out using JoinMap (version 4.0) for each parent separately. The cross pollinator (CP) population type code was used to score the genotypic data. The Kosambi (1943) mapping function was used to convert the recombination fractions into centiMorgans (cM). Linkage between two markers was determined significant in two-point linkage analysis using a likelihood odds (LOD) ratio of 7.0. The linkage groups that did not have more than three markers were omitted from the map. Linkages were recombined within each parent separately using the module ‘combine groups for map integration’ in JoinMap, which led to seven homology groups for each parent. At this stage, assuming that the SSR alleles are from a single locus, then the polymorphic SSRs acted as allelic bridges. The linkage maps were then finally aligned into a single integrated linkage map on the basis of a subset of the common markers that were present in both recombined parental maps.

## Results

### Polymorphism and marker segregation analysis

Out of the AFLP primer combinations, 10 revealed polymorphisms that were suitable for assessing the 189 F_1_ progeny as they were highly conserved. The size of the AFLP fragments ranged from 51 to 559 bp. Three cases of polymorphism were considered, which were fragments present in ‘Yunzheng Xiawei’ and absent in ‘Sun City’; fragments present in ‘Sun City’ and absent in ‘Yunzheng Xiawei’; and fragments present in both parents and segregating in the population. As shown in Table 1, 206 polymorphic amplification markers (98 were specific to ‘Yunzheng Xiawei’ and 108 were specific to ‘Sun City’) were suitable for use out of the 439 fragments in total. The exact numbers of the markers used in the different steps (step 1–preliminary parental linkages, step 2–recombined parental maps and step 3–final integrated map) of map construction are shown in Table 2.

**Table 2:** **Number of AFLPs and SSRs positioned on the genetic linkages**.

**Type**	**Total markers**		**Polymorphic markers**		**Markers on the preliminary parental linkages**		**Markers on the recombined parental maps**		**Markers on the final integrated map**
	**Y**	**T**	**Y**	**T**	**Y**	**T**	**Y**	**T**	**Y × T**
AFLP	183	256	98	108	49	38	38	18	53
SSR	517	491	329	307	249	217	171	148	242
Total	700	747	427	415	298	255	209	166	295

Y represents ‘Yunzheng Xiawei’ and T represents ‘Sun City.’

In addition to the AFLPs, a set of 199 SSRs were informative for map construction out of 697 primer pairs, which in total amplified 517 fragments for the female parent ‘Yunzheng Xiawei’ and 491 fragments for the male parent ‘Sun City.’ Among these amplified products, 329 and 307, respectively, were polymorphic with 249 (76%) and 217 (71%) able to be positioned successfully (step 1) on the preliminary parental linkages for ‘Yunzheng Xiawei’ and ‘Sun City.’

When different lineages have common SSRs, it is possible to generate a combined map. A total of 375 polymorphic markers were generated when the two parental maps were considered in step 2. Among these markers, 209 originated from ‘Yunzheng Xiawei’ and 166 originated from ‘Sun City.’

Of these, 74 common SSRs existed in both recombined parental maps, and these were used to construct the final integrated map. Finally, in total, 242 polymorphic SSRs and 53 AFLPs were identified (Table 2).

On the final integrated map, 108 pairs of newly developed EST-SSRs (Table S1) provided 149 polymorphic markers, accounting for approximate 50% of the markers. However, they were unevenly distributed, with the proportion ranging from 25% (LG 5) to 61% (LG 2). Detailed information, including the primer sequences, for the EST-SSRs is available if requested. The statistical data in Table 3, including map density, average distance between markers and largest gap between markers, showed that the density of the map steadily increases.

**Table 3:** **Distribution of markers on the recombined parental maps, final integrated map and linkage group statistics**.

**Linkage group**	**AFLPs**	**Number of the available SSRs**	**Number of newly developed SSRs**	**Number of markers exhibiting segregation distortion (*P* < 0.05)**	**Map length (cM)**	**Map density (markers/ cM)**	**Average distance between markers (cM)**	**Largest gap between markers (cM)**	**Original linkage distribution in Figure S1**
**MATERNAL LINKAGE GROUPS-‘YUNZHENG XIAWEI’**									
A1	11	18	29	1	109	0.53	1.88	13.0	Y1 + Y2 + Y3
A2	3	2	6	0	64	0.17	5.86	30.9	Y5 + Y7
A3	1	4	5	2	92	0.11	9.19	22.3	Y6 + Y9
A4	4	14	16	1	116	0.29	3.42	13.5	Y16 + Y17
A5	7	9	6	3	112	0.20	5.11	18.0	Y8 + Y10
A6	9	6	11	3	135	0.19	5.20	25.0	Y18 + Y19 + Y21 + Y22
A7	3	17	28	16	109	0.44	2.27	26.8	Y12 + Y13 + Y14 + Y15
Total	38	70	101	26	737	–	–	–	–
**PATERNAL LINKAGE GROUPS-‘SUN CITY’**									
B1	4	19	36	2	175	0.34	2.97	26.6	T1 + T2 + T3
B2	4	5	18	3	140	0.19	5.19	47.6	T4 + T5 + T7 + T14
B3	4	0	3	0	56	0.12	8.07	22.0	T10 + T24
B4	3	12	15	3	172	0.17	5.73	21.8	T6 + T23 + T25 + T27
B5	0	5	3	2	53	0.15	6.60	23.5	T11+T28
B6	2	5	8	0	76	0.20	5.06	11.6	T8 + T9 + T15
B7	1	7	12	0	79	0.25	3.97	26.2	T18 + T19 + T20 + T31
Total	18	53	95	10	752	–	–	–	–
**INTEGRATED LINKAGE GROUPS**									
LG1	9	7	15	3	130	0.24	4.19	24.1	Y18 + Y19 + Y21 + Y22 + T16
LG2	7	24	48	17	148	0.53	1.87	17.2	Y12 + Y13 + Y14 + Y15 + T1 + T2 + T3
LG3	3	5	8	0	65	0.25	4.07	25.3	Y25 + Y26 + T8 + T9 + T15
LG4	5	9	18	2	95	0.34	2.98	11.6	Y5 + Y7 + Y6 + Y9 + T18 + T19 + T20 + T31
LG5	7	11	6	3	161	0.15	6.71	39.5	Y8 + Y10 + T11 + T28
LG6	15	19	33	3	129	0.52	1.93	8.1	Y1 + Y2 + Y3 + T4 + T5 + T14
LG7	7	18	21	3	146	0.31	3.18	16.2	Y16 + Y17 + T6 + T23 + T25 + T27
Total	53	93	149	31	874	–	–	–	–

The exact number of distorted markers on the recombined parental maps and the final integrated map are shown in Table 3. Of the markers on the recombined parental maps, 36 (12%) did not follow a standard Mendelian segregation (*P* < 0.05), but they were still maintained during the linkage map construction.

### Linkage map construction

A total of 427 and 415 polymorphism markers (SSR and AFLP) were employed to build maps, which were construction by performing three steps. First, the preliminary parental linkages were constructed and consisted of 26 and 32 groups, respectively, which putatively corresponded to the 28 tetraploid level rose chromosomes. These linkages covered a total length of 1966 cM in the maternal ‘Yunzheng Xiawei’ and 1882 cM in the paternal ‘Sun City,’ and respectively had average chromosome lengths of 75 and 59 cM (Figure S1). The linkage distance spanned by individual linkage groups ranged from a low of 2.7 (Y7, Y20) to a high of 183 cM (Y16). There were also several linkage groups, from both maps, that were small and contained less than three markers.

**Figure 2:**
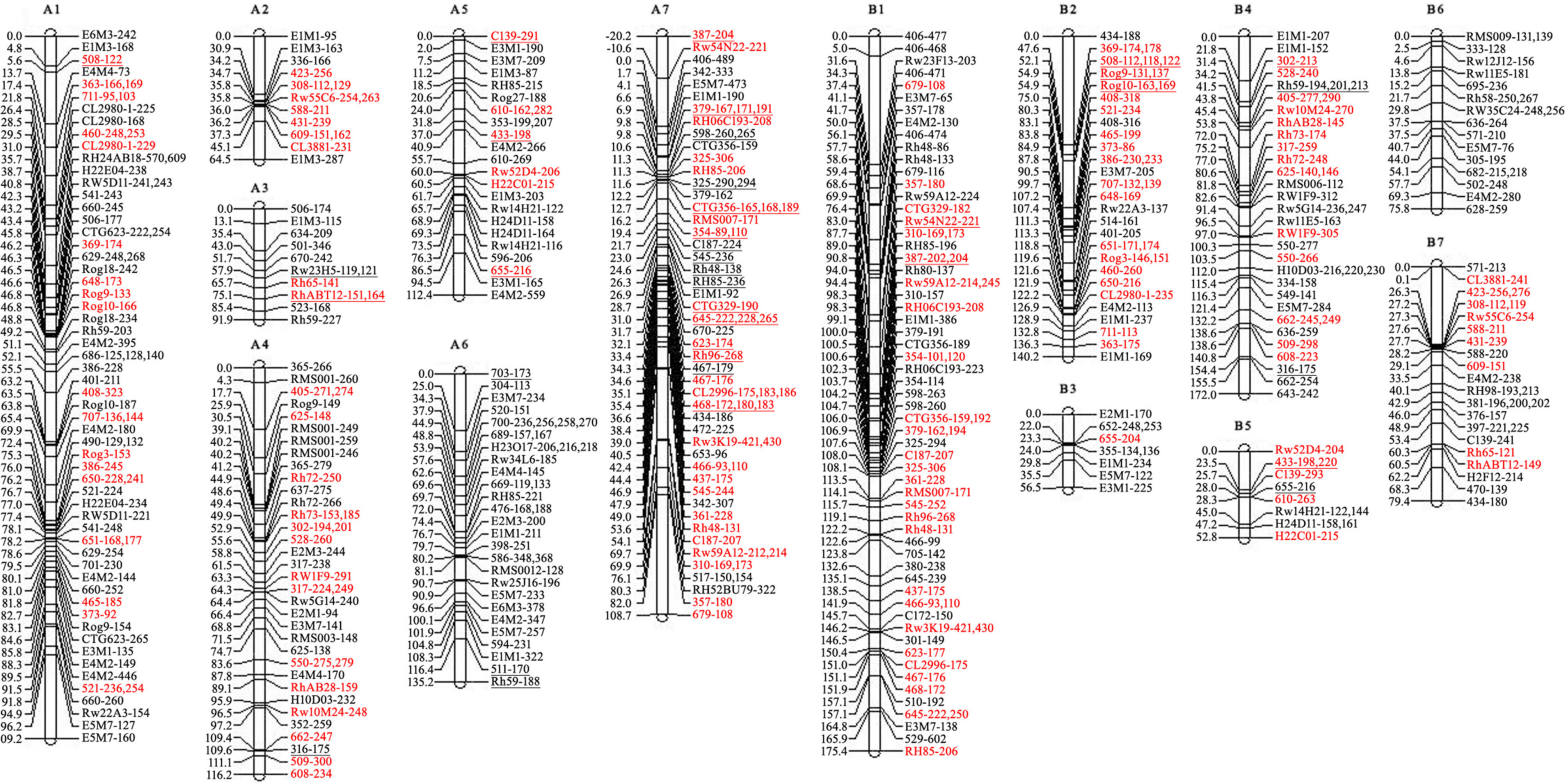
**Recombined parental maps**. A1–A7 for ‘Yunzheng Xiawei’ and B1–B7 for ‘Sun City.’ Map distances are shown in cM on the left of each linkage group. The distorted segregating markers are underlined. Common markers between the two parental maps are marked in red.

Then the recombined parental maps with the common SSRs were drawn, which led to seven homology groups for each parent (Figure 2). The map lengths were 737 and 752 cM, with 209 and 166 marker positions and average distance between markers of 3.5 and 4.5 cM for ‘Yunzheng Xiawei’ and ‘Sun City,’ respectively.

**Figure 3:**
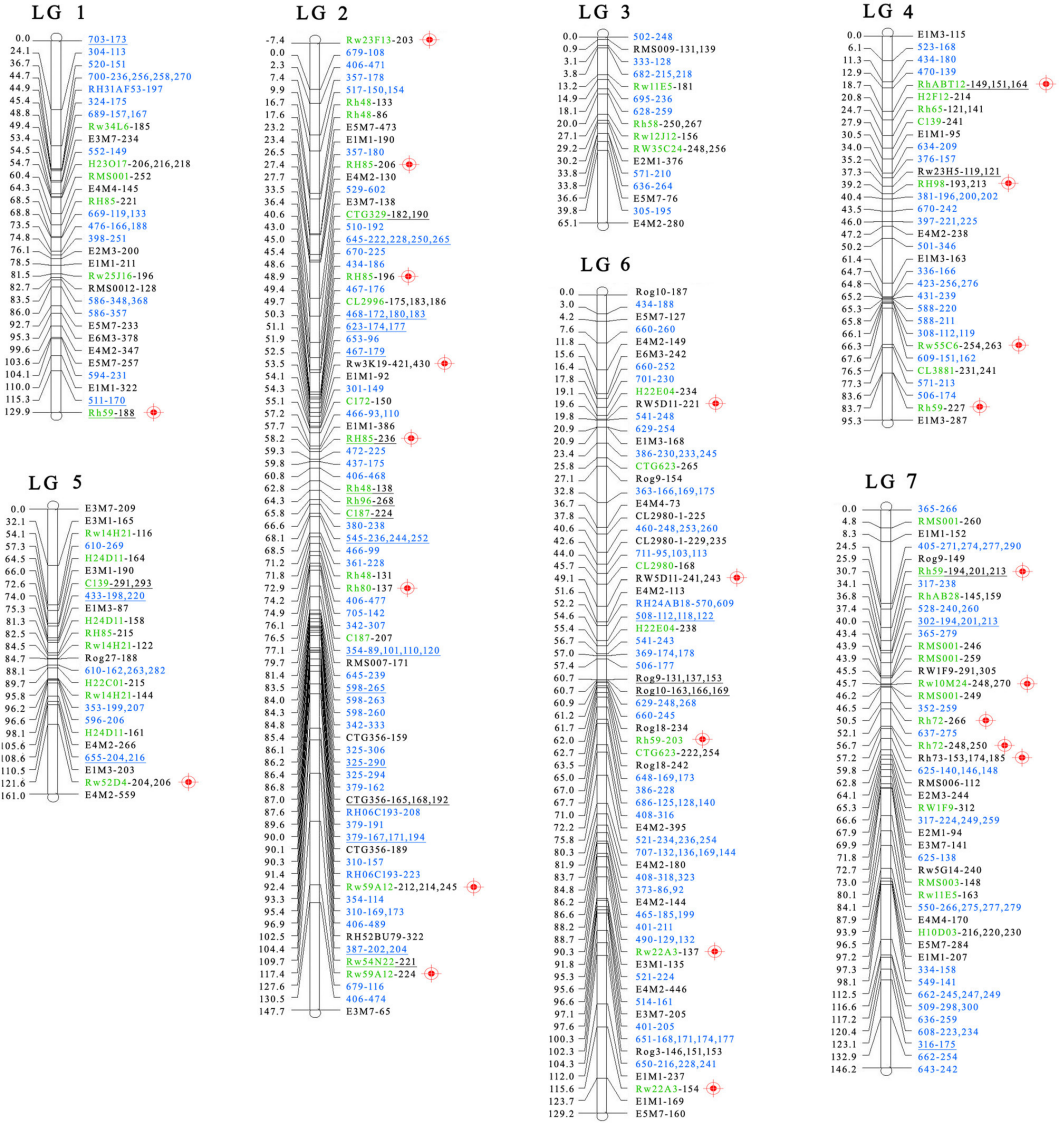
**Final integrated map for ‘Yunzheng Xiawei’ and ‘Sun City.’** Map distances are shown in cM on the left of each linkage group. The distorted segregating markers are underlined. The markers derived from the newly developed EST-SSRs are marked in blue. Common markers between the final integrated map and the integrated consensus map (ICM) (Spiller et al., 2011) are marked in green. Common markers with the K5 map (Koning-Boucoiran et al., 2012) are marked with a red anchoring point 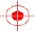.

Finally, the two maps were combined to form a single integrated map with 74 pairs of common SSRs available for both recombined parental maps. The markers on the integrated map have a similar order as to when they were on the separate parental maps. The final map was aligned with seven integrated linkage groups, which had a calculated total length of 874 cM and 295 polymorphic markers (Figure 3).

## Discussion

### Mapping population

When constructing a genetic linkage map, the genetic background of the parental material, size of the segregating population and features of the molecular markers selected must be taken into account. In roses, the previously constructed genetic linkage maps have focused on the diploid level while mapping at the tetraploid level lags behind (Gar et al., 2011; Spiller et al., 2011; Koning-Boucoiran et al., 2012). In this research, a tetraploid population consisting of nearly 200 individuals was selected to provide more detailed and reliable information for the tetraploid map. Comparisons between the maps (diploid and tetraploid, tetraploid and tetraploid) will enable more reliable marker-trait associations to be determined.

In addition, all the parental material for the existing tetraploid mapping populations was selected from modern rose cultivars that are closely related, which can cause a failure for downstream breeding applications. In this research, ‘Yunzheng Xiawei,’ a Chinese traditional rose cultivar was used to enlarge the relatively narrow genetic background for the rose map construction, which may help to cover more genome regions and fill gaps within the linkage groups.

### Marker diversity and segregation in mapping population

Currently, SSRs, which have been proven to be effective and highly polymorphic markers, are widely applied for the construction of genetic linkage maps in crops, trees, fruits, and flowers. When a map has been constructed using SSRs it is possible to integrate information from previously produced genetic linkage maps due to anchor points. AFLPs can generate a large number of polymorphic markers without any prior knowledge of DNA sequences. AFLP fragments are related to unique positions in the genome (Vos et al., 1995), so they are complementary with SSRs (Julier et al., 2003). The two kinds of efficient polymorphic markers can capture different information in a genome due to dissimilar mutation rates and non-uniform linkage disequilibrium (LD) distribution among the chromosomes (Du et al., 2013).

In the final integrated map produced by this research, a total of 53, 93, and 149 evenly distributed polymorphic markers derived from AFLPs, available SSRs and newly developed EST-SSRs, respectively, were used to construct it. The AFLP s help to fill gaps, while the SSRs will enable integration of information from other sources. The EST-SSR based linkage groups can be further explored between the genotypes and phenotypes to identify QTLs, which are directly tagged to functional genes (Lu et al., 2012). In addition, once the rose genome has been fully sequenced, these markers will form the basis of a method to develop a high density genetic map that has SSRs distributed throughout the genome.

### Mapping in tetraploid rose

We obtained longer and more saturated maps with the AFLPs and SSRs in tetraploid cultivated roses than in the most recently published map (1081 and 1225 cM for the whole genomes in P540 and P867 over 28 linkage groups, respectively) with AFLPs, NBS and SSRs (Koning-Boucoiran et al., 2012). The preliminary map covered about 97% of the rose genome at the tetraploid level. The existence of minor linkage groups and the unlinked markers indicates that there are many large gaps with few markers (Chen and Chen, 2010).

Compared with Gar's tetraploid parental maps (2011), which covered 632 cM (259 markers) for FC and 616 cM (210 markers) for GG, the recombined parental maps shows that the map constructed in this work has greater coverage, but the marker density has decreased (from 2.9 to 4.5 cM). The differences may be due to the size of the mapping population, selected markers or software used to construct the map.

There were 74 markers that were common between ‘Yunzheng Xiawei’ and ‘Sun City,’ and from these 295 polymorphic markers could be identified to construct the map. The map length spanned 874 cM with a mean intermarker distance of 2.9 cM. The linkage groups were covered well by the markers that were regularly spaced along the homologous chromosomes, thus the genomic coverage was considerably improved by the addition of the newly developed EST-SSRs. For example, the length increased to 129 cM (by 93%) for LG 6, which had the lowest coverage when considering the integrated consensus map (ICM) and K5 map. However, gaps larger than 20 cM were found to be located toward the ends of LG 1, LG 3, and LG 5. The regions of low marker density lead to different LGs forming and may be associated with the conserved genomic regions with limited genetic variability (Shahin et al., 2011) rather than population structure or marker type. This could also be caused by the hotspot terminal regions within a genotype or population that have higher levels of recombination frequency than other areas of the chromosomes (Xu et al., 2011; Chancerel et al., 2013; Sun et al., 2013).

### Map comparison with SSRs

A comparative analysis of the integrated map with a recently published tetraploid map (Koning-Boucoiran et al., 2012) revealed that all (Rw23F13, Rh80, RH(h)85, Rw59A12, Rw10M24, Rh72, Rh73, Rw52D4, Rh59, RhABT12, RH(h)98, Rw55C6, and Rh58) but three (Rw22A3 (LG 6-A4), Rw5D11 (LG 6-A4&B4) and Rw3K19(LG 2-A6) features were gathered in the same groups over the 16 common SSRs.

In addition, a comparison between the integrated map and the diploid ICM (Spiller et al., 2011) showed that 42 common markers were located in the same linkage group (Table 4 and Figure 3). The linkage groups could be well-matched between the common markers. The order of these bridge markers was consistent between the two individual maps in most cases; however, some marker order discrepancies and differences in the calculated map distance were observed. The quality and accuracy of marker order in such maps depend on numerous factors, including segregation distortion, population size and scoring errors (Jairin et al., 2013). On the integrated map, 10.5% of the markers were distorted, and they were not evenly distributed on the map. Of the 31 distorted markers, 17 were clustered on LG 2. This phenomenon has been widely reported, and is due to both biological factors and statistical bias or errors in genotyping and scoring (Gao et al., 2008; Chen and Chen, 2010; Lu et al., 2012). Another factor linked to the accuracy with which recombination events can be detected is the size of the mapping population (Hackett et al., 1998; Jairin et al., 2013). Compared with the diploid ICM (Spiller et al., 2011), we used a relative small population size, thus inaccurate marker order may have occurred due to inversions and low marker distance.

**Table 4:** **Comparison between the tetraploid ‘Yunzheng Xiawei’ and ‘Sun City’ linkage map generated in this research and the diploid integrated consensus map (ICM) (Spiller et al., 2011)**.

**Linkage group code**	**Number of common markers**	**Names of common markers**
LG1	3	Rw25J16, Rw34L6, H23O17
LG2	11	Rw23F13, Rh48, Rh80, RH85, CTG329, CL2996, C172, Rh96, C187, Rw59A12, Rw54N22
LG3	4	Rw11E5, Rh58, Rw35C24, Rw12J12
LG4	8	Rh59, H2F12, RhABT12, C139, Rh98, CL3881, Rw55C6
LG5	5	RMS001, Rw14H21, H24D11, H22C01, Rw52D7
LG6	4	Rw22A3, CTG623, H22E04, CL2980
LG7	8	Rw10M24, RMS003, Rh72, Rh73, Rw5G14, Rw1F9, RhAB28, H10D03

In addition, several primer pairs amplified different alleles that were mapped to different positions on the same (310, 342, 354, 357, 406, 466, 467, 645, 679, C187, CTG356, RH06C193, Rh48, and Rw59A12 on LG 2; 610, H24D11 and Rw14H21 on LG 5; 386, 401, 408, 521, 541, 629, 660, CL2980, CTG623, H22E04, Rw22A3, Rog9, Rog10, Rog18, Rw22A3, and RW5D11on LG 6; 365, 662, RMS001, Rh72, and RW1F9 on LG 7) or even different linkage groups (434 on LG 2, 4, and 6; 506 on LG 4 and 6; 571 on LG 3 and 4; 636 on LG 3 and 7; 670 on LG 2 and 4; C139 on LG 4 and 5; Rh59 on LG 1, 4, 6 and 7; Rh85 on LG 1, 2 and 5; RMS001 on LG 1 and 7; Rog9 on LG 6 and 7; Rw11E5 on LG 3 and 7). The mapping of different alleles to positions larger than 10 cM on different LGs could be attributed to the amplification of different loci. SSRs with these patterns were also observed in the previous rose maps. For example, SSRs C139 and H9B01 were detected on the linkage map of H190 and *R*. *wichurana* (Hibrand-Saint Oyant et al., 2008), SSR Rh59 was found on both LG 4 and 5 on ICM (Spiller et al., 2011) and several multi-allelic SSRs were successfully used to assign linkage groups to the seven basic sets of LGs on the K5 map (Koning-Boucoiran et al., 2012). The high information content and transferability of the SSRs not only provides an important tool for comparative alignment and integration of related maps (Li et al., 2010), but also results in the differences between the different populations studied (Koning-Boucoiran et al., 2012). Nevertheless, these problems will be eventually solved via the fast development of next generation sequencing technology, and then a more accurate marker order can be acquired by means of map validation when a draft genome sequence for rose becomes available.

In summary, common SSRs were distributed within the different linkage groups in the present study, and these can be used as anchoring points in the future, while the newly developed EST-SSRs complement them well. In the future, the anchor points and complementary markers will enable the gaps that are present to be significantly closed. In addition, more markers are needed toward the ends of each linkage group and in general to obtain a density close to saturation. Once a dense consensus map has been produced it will be a valuable tool in many genetic and genomic applications, especially for fine-scale mapping and map-based cloning of trait-controlled genes in roses.

### Conflict of interest statement

The authors declare that the research was conducted in the absence of any commercial or financial relationships that could be construed as a potential conflict of interest.

## Supplementary material

Figure S1 **Preliminary linkage groups of the maternal ‘Yunzheng Xiawei’ and the paternal ‘Sun City.’** Map distances are shown in cM on the left of each linkage group. Distorted segregating markers are underlined.
