# Supplementary figures and images for: Filling gaps with construction of a genetic linkage map in tetraploid roses
Source: Front Plant Sci. 2015 Jan 13;5:796. doi: 10.3389/fpls.2014.00796 (PMC4292389; doi:10.3389/fpls.2014.00796)

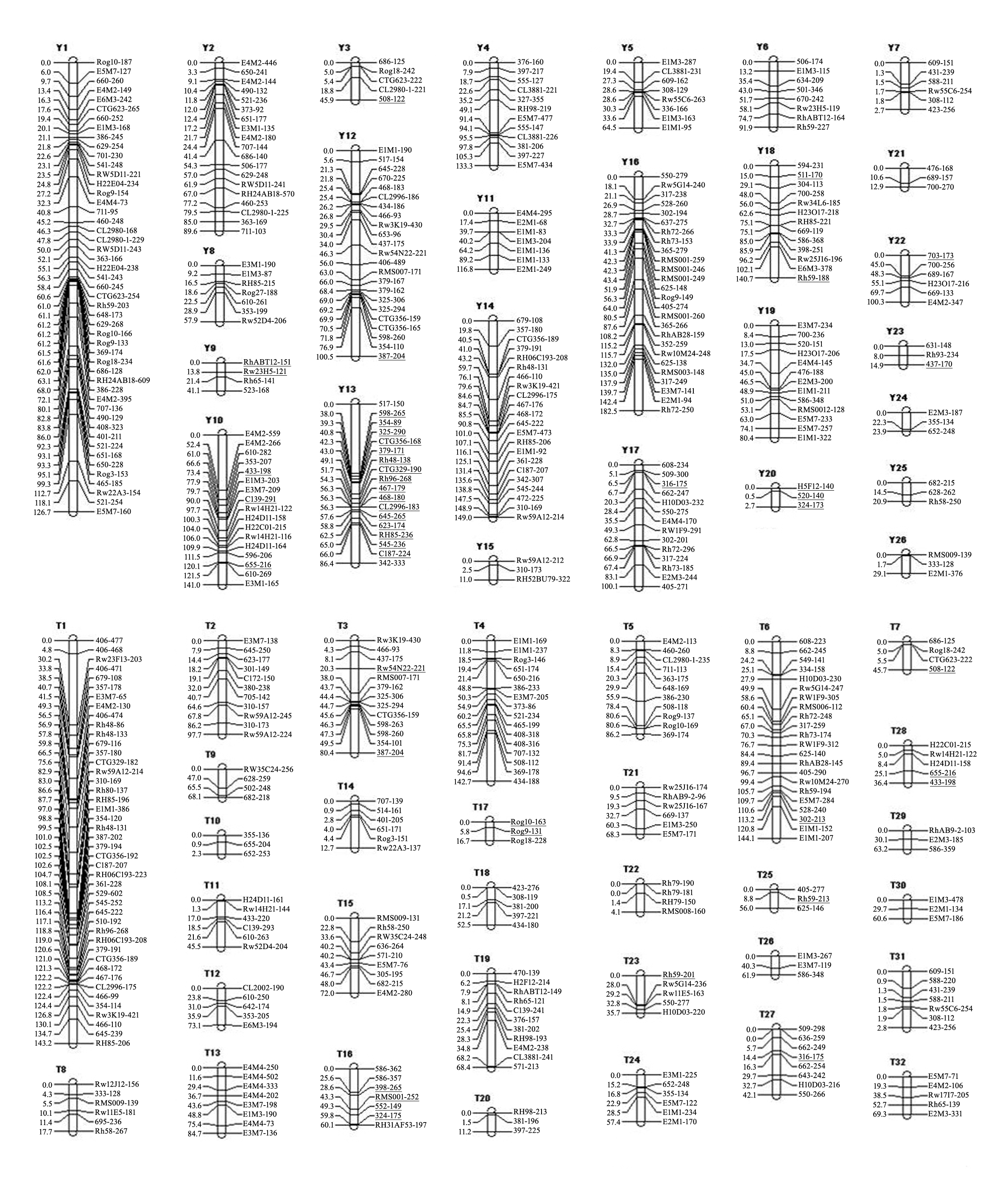

Supplement: Figure S1 — Preliminary linkage groups of the maternal ‘Yunzheng Xiawei’ and the paternal ‘Sun City.’ Map distances are shown in cM on the left of each linkage group. Distorted segregating markers are underlined. [file Image1.JPEG]
