# Supplementary material for: Filling gaps with construction of a genetic linkage map in tetraploid roses
Source: Front Plant Sci. 2015 Jan 13;5:796. doi: 10.3389/fpls.2014.00796 (PMC4292389; doi:10.3389/fpls.2014.00796)
Supplement: Supplementary file 2 [file Table1.DOC]

**Supplementary table 1**

Characteristics of the 108 newly developed pairs of EST-SSRs positioned on the final integrated map.

| SSR name | SSR motif | Tm (°C) | Forward（5’→ 3’） | Reverse (5’→ 3’) |
| --- | --- | --- | --- | --- |
| RH06C193 | (AGA)_4_ | 50.7 | GAGGAGTGGGTGGAAGAT | CAGTAAATCAAGGGACAAAA |
| RH31AF53 | (TGCTGT)_3_ | 53.6 | TGGAATGGAGGCTACTGC | TGTTCTCACGGTGGCTGT |
| RH24AB18 | (AGACTC)_3_ | 49.0 | ATCGCCCTTTGATATTGT | CGTTGGCTTGTTGTTGTA |
| 301 | (AGA)_7_ | 59.4 | GACAACACCAACTAGAACTTGAGC | GCTCAACAGCAACAACCTCA |
| 302 | (CT)_16_ | 59.9 | CAAAGTCCCTTCCATTTCCA | TGTTGGATTTGCCTCCTTTC |
| 304 | (GA)_16_ | 59.0 | GGGTCGGACTGGAAATAAGA | CTTTCTCTCTGATCGCCTGC |
| 305 | (CAAGAA)_6_ | 59.9 | GATGCTGAGCAGAGCAACAG | GTTGCTCGGGTTGTGAAACT |
| 308 | (AGA)_9_ | 59.6 | AAATTAGGTCTTGGAAATTTGGG | ATCACCTTCTGCTGAAACGG |
| 310 | (ATCT)_7_ | 57.9 | GGAGTCGAGATCACCTGAGA | GGCTGGAGAAGCTGAAATTG |
| 316 | (GAG)_8_ | 60.1 | GTAGACGAGGATCCGAACCA | ACTCAGCAGGCCGCTTATTA |
| 317 | (TC)_14_ | 60.2 | ATTCTCAAATCCAGCGAACG | CCGAAATTGCCAGATCCTAT |
| 324 | (GA)_7_ | 60.4 | AACAACTGCGAAAGCAAAGG | GTTGTCGACGATGCTGAGAA |
| 325 | (CTG)_5_ | 60.0 | GAAGATGAGGCAGAACAGGC | AAAAGAGGCAAAAGCCCAAT |
| 333 | (CTT)_5_ | 59.4 | GGCTGCTACTACAACTTCCCTC | GATCTGCCTCGCCTTAACTG |
| 334 | (AAAAC)_3_ | 59.0 | TCTTTCAGGCCTCTCACCTT | AAACGACGAAGCACCTCACT |
| 336 | (TC)_9_ | 59.9 | CAAACGAAACCCTCTGCTTC | GACGATGCATTTGGTGTGAC |
| 342 | (GA)_13_ | 58.5 | GATTAAAGAATTGAGCAAACACTTG | CCACTGTGTCCAGAAGCTCA |
| 352 | (ATTTGG)_3_ | 59.5 | GGAGATCGAAGGAAGCTTGA | CCTCATTGCCCTGGTTCTTA |
| 353 | (TC)_7_ | 59.7 | CGCCCTAGTCTCCTCTCTCTC | CTCAAGCTGAAGCTCGGAGT |
| 354 | (TTGAAG)_3_ | 60.0 | TAGAGCCGAAACCAGTTGCT | GAGGCGTGAACGAACTAAGC |
| 357 | (AG)_15_ | 58.9 | ATCATTAAAGGTTGGTCGCC | CTTTGGGCTGTTTCACATCA |
| 361 | (TCT)_7_ | 60.0 | TGTGCCTCGAGAGGTTTCTT | TTGGACTTGAAGTTGGAGGG |
| 363 | (AAG)_6_ | 60.0 | GAAAGCTTGAAGGTCGAACG | GTTCTCGAACTTGTCGAGGC |
| 365 | (TCA)_5_ | 60.0 | GAGAAAGTTCGCGTCTTTGG | GAGGAGAGAGACGCTTGCAG |
| 369 | (AGAA)_4_ | 60.2 | TGCCACACACTTGAGTCCAT | AAACAACATGCTACTCCGGC |
| 373 | (CT)_10_ | 58.9 | ACAAACTTCGCGATTCCTCT | AGTTCCAGACGTTGGAGTGC |
| 376 | (TTC)_5_ | 60.0 | TCCCCCAAAACCAAAATACA | TCCTCGTGGAAGAGCAAAGT |
| 379 | (GGA)_6_ | 60.0 | GTCATCAAGGAGGACCAGGA | GATCAGCGACCACCATGTC |
| 380 | (TCA)_5_ | 59.9 | CTTCACCACCCCTTCAAAAA | GGATGGCAACTTTGTTCGAT |
| 381 | (TC)_11_ | 60.2 | GACCCTCTAAGCGATAGGGC | GCAACAAGAACCAGCACAGA |
| 386 | (GGA)_5_ | 60.1 | TCTTCCCCTAACGTCGTGTC | CTCATCATCACCACCACCTG |
| 387 | (CT)_9_ | 58.7 | GCACTCTTGACGTTGTCCAT | GTCAATGTAGTCCGGTTCGG |
| 397 | (CT)_14_ | 59.9 | GGCCTAGCAAAGCAACAAAC | AGTGGAGGGCAGTCTCTGAA |
| 398 | (CT)_8_ | 61.0 | GCAAAAGCACCAACCAAAAA | CTCGATGTGAGGGATGACCT |
| 401 | (AATCC)_3_ | 60.0 | CCTCCTTCTCAAACTCGCAC | TCTGCTTTCCTTTGCTCCAT |
| 405 | (ATG)_5_ | 59.9 | CAGCGAAAAGAACAAGGACC | CAGAAGCTAATAAATTAACAATCACCA |
| 406 | (CT)_9_ | 61.8 | GGAGCCTCCACTACCCCC | CACAGTCTTCTTGTTCGCCA |
| 408 | (GA)_9_ | 58.8 | GGAGTGAAACAAGGGAGAGTG | GCTTGTTGGTCTCGAACCTC |
| 423 | (CT)_7_ | 59.8 | CGCTGACCCGATAGAAAGAC | CTGACGGCGAGAAGAAGATT |
| 431 | (CAG)_5_ | 59.8 | CTTTCGAGGGTGTTTTCGAG | ATCTTCGGATTGCTCACCAC |
| 433 | (AG)_8_ | 59.5 | CGAGATGACCTGTAATTCTATCCA | TCTTCATGATCCTGCTGACG |
| 434 | (AG)_7_ | 60.0 | CTCTTTCGGGTCATAGCTCG | GTGGACGTAGTCTCCCGCTA |
| 437 | (GCCGGA)_3_ | 59.6 | CTGAGGAACACGAGAAGCCT | CAACCTCTTCCTCAGCCTTG |
| 460 | (TCT)_6_ | 60.0 | CGCTTCCTCCATCAATCAAT | ATCTGCACCTGTCTCTGGCT |
| 465 | (CTC)_5_ | 59.9 | ACGCAGCAAACACAGCATAC | TGTTCGATGTACTGCAAGGC |
| 466 | (CT)_11_ | 59.9 | TAAGCTTAAGCCGTGCCATT | AAACTCGACATCGGAGAAGC |
| 467 | (CGA)_5_ | 60.0 | GTACGCTCTCTGGTCTTCGG | CCCATGTCTCTGGCTATGGT |
| 468 | (CCG)_6_ | 60.0 | ACCATAGCCAGAGACATGGG | GGGCAGAGAAGAAGTTGACG |
| 470 | (TCT)_5_ | 60.1 | AACCCAACGTGCTTCTTTTG | GGTCTTCATTGTTGTGGGCT |
| 472 | (TCT)_7_ | 60.0 | GCACCCTAACAGACCCAAAA | TCCCATCTTCTCTCTGAGCAA |
| 476 | (CT)_9_ | 60.4 | GGGAGTGATGTTTGTGTGAGG | GAATTCCGAGTCCAACGAAA |
| 490 | (TCT)_6_ | 60.0 | ACAACCAACCCAAGAACTCG | TCCCAGCTTCAGTCTCACCT |
| 501 | (GAA)_12_ | 59.4 | GACAGACAGAGCTGCAGACAA | GTTGAAAGCCCCAAAACCTT |
| 502 | (TC)_7_ | 59.5 | CTGTGTGACAGTGCATTCTGAG | TTCAAGATGGACATGCCGTA |
| 506 | (AAG)_5_ | 59.2 | AAGAAAGGAAAAACTGATAGGGC | AATGACTAGATCAACCGGCG |
| 508 | (AG)_9_ | 59.6 | GAACTCAAGGAGGGATAACGAA | ATCTCTCATCGGAATGTGGG |
| 509 | (CAC)_5_ | 60.0 | CAACTGGGTTGGGTCAGTCT | TCAAATGTACCTTGCGCTTG |
| 510 | (AAG)_5_ | 60.1 | AGAGGTTTAGGGCAGCCATT | GCGAATGATGGTGGAGAGTT |
| 511 | (AGA)_5_ | 59.1 | GAAAGCCAAAAGCTCACACA | GATGATGAGGGTGAGGAGGA |
| 514 | (TC)_7_ | 59.1 | AATCCCCAAACCCTAACCTC | AGCTCCGGCTAAGGATTCTC |
| 517 | (AACCG)_3_ | 60.0 | TGCACCGAAAAATGGTGTAA | AAAACGTTAAGGGTGGGAGC |
| 520 | (AAG)_5_ | 60.2 | CACAGAAACGAAGCGCAGTA | GCTCGAAGAAGTCCTGGATG |
| 521 | (GA)_8_ | 60.5 | GTTCCAGCAGCACTCCAAGT | AGAGGGGATTAGCTGCACTG |
| 523 | (CATTCA)_3_ | 59.5 | GCAACAAGGACCCCTTCTTAT | CCATCCATCGGAGAAGAAAA |
| 528 | (CTG)_7_ | 60.1 | ACAGGCCTCTGTTCACCATC | GGATGGGACATCCAAGTCAT |
| 529 | (TCT)_5_ | 58.4 | CAGAAAAGTACTGTTGGTTCTTCC | TCCTTCAAGCTAAGGCCAGA |
| 541 | (AG)_7_ | 59.7 | CTACTCCAATGTCCGCTTCC | GTTGGAGAAGAAGCCGTGAG |
| 545 | (TC)_12_ | 60.6 | GCAAACCTCCCGTCTTTTCT | TAGTTCGTCGGAGGAGAGGA |
| 549 | (GAA)_5_ | 59.9 | TGGAGGAGAAGGAGAAACGA | ATGTACTTGTGGCTCGGACC |
| 550 | (GA)_7_ | 60.4 | CTCCAGCGGTTCTGTTCTGT | ACCAAGAGGAGAGGCAGTGA |
| 552 | (TCC)_6_ | 59.7 | ATTTCCAAAAATGGCACCAC | GTTGAAAGTGGAGAGCTCGG |
| 571 | (AG)_8_ | 59.2 | AAAGTAAATAAAACAAAAACAGGCG | GGGTCTGGTGGAGTAGACGA |
| 586 | (TCT)_7_ | 60.0 | TCCTTGATGCCTGCTTTCTT | GGTGGAGTTCTTGTTGGCAT |
| 588 | (CTT)_5_ | 59.8 | CCCAGAAAAACCCAAAACAA | TCGAAATTGGTGACGTTCAA |
| 594 | (CT)_8_ | 59.6 | GGCCTGAGGTGCTTCTTCTA | ATGAGACCAATCTGCCAACC |
| 596 | (AGG)_5_ | 60.6 | CGAGGAAAAACCCAAAATCC | TGGAAGCAAGAAAAGGCAGT |
| 598 | (CTC)_6_ | 59.8 | GAGAGAGGAAAAGGGTGGCT | GGCACATGTTTGGTGAGATG |
| 608 | (GGAGCA)_3_ | 60.1 | TCCTCGACAACCCTAACGTC | ACGGACTTTTGTTGAATGGC |
| 609 | (CT)_18_ | 59.6 | GGGCATTGTCCATCTCTCTC | CTGTCTCCGTGGGTTATGGT |
| 610 | (CT)_18_ | 59.9 | ACACCCGAAACTGAAACACC | ATAGGAAATGGTGCGGTGAG |
| 623 | (CT)_10_ | 60.1 | CATAACCCGGATTCCACAAC | CGAGATCAGCAGCACCATTA |
| 625 | (TC)_8_ | 59.0 | GGCGTCTCTCACATCTCAAA | AAGATCTTCTCTCCGGCCTT |
| 628 | (AAG)_5_ | 60.1 | AACGAACGAGTTGGAGTTGG | ATTGCACAGCATTATCAGCG |
| 629 | (CTT)_7_ | 59.7 | CACGAGCTCTCTCTCCCCTA | TTGGTCTGTGAAGTGGTGGA |
| 634 | (CT)_9_ | 59.0 | CACGAGCTGAACTGACCACT | TGCTAGGGAACTGGAGGAAA |
| 636 | (CCACAA)_4_ | 59.8 | CATTTTCCCAACAAGTCGGT | AGCTGGTGGAGTTGCTCTGT |
| 637 | (TTGATT)_3_ | 60.1 | GCCGTAATTCGTGGAAAGAA | ATGCCACCAGAACCTTGAAC |
| 643 | (CCT)_5_ | 60.0 | AAACTTGCCACCATGTCCTC | AACTCATCGGTGATCTTGCC |
| 645 | (TC)_11_ | 60.0 | TTGCGTAGGAGAGCTCCATT | TTCGATTCAGACCCAACTCC |
| 648 | (CT)_8_ | 60.5 | CCTAAAGCTTAAGCCCCCAA | GCAATAGACTTGGCAGCCTC |
| 650 | (CT)_11_ | 60.8 | ACGAGCCTTTGTTGCCCT | CCTGTTGAGTCGTGCTCAGA |
| 651 | (CAG)_5_ | 60.2 | TCTGAGCACGACTCAACAGG | AGGCATGTAATGCTGTGGGT |
| 653 | (GCC)_5_ | 60.0 | CAACCCGCCAATACTGAACT | TGGTCTGATGGGGTAGAAGG |
| 655 | (GA)_9_ | 60.0 | CCAACCCTTTCTCTTCCTCC | AACAAAACAAAGCTGGACACC |
| 660 | (CATTT)_4_ | 60.5 | CCCGCTTTCCTTCTTAGCTC | TGGTTGTGGTTGTGTCCTGT |
| 662 | (AG)_11_ | 59.9 | GCTTTGTCTGTGATGGCGTA | TCACTTTGAGGGCAAATTCC |
| 669 | (GA)_7_ | 60.1 | GACGGGTAGAAGCGGTGATA | CCAGACTCGAATCCGTCATT |
| 670 | (CT)_17_ | 59.7 | TCATTAATGCCTCCTCCTTCTC | TTTGGGAATCTCCCTCTGTG |
| 679 | (AT)_12_ | 60.0 | GAGTGGTCTATGGCCGGTAA | AGTGCGCACAAATCAATCAG |
| 682 | (TC)_10_ | 59.7 | TTCTTGAGCTAAAAGTGCATCG | CAGATCCAAACCGAACCCTA |
| 686 | (GAA)_8_ | 59.9 | CACGAGTGTCACTGTTGCCT | AGAATTGGCTTAGCTTGGCA |
| 689 | (CT)_10_ | 60.0 | TTTCCCCATTTCCCCTAAAC | GTTGTTGCTGTTCCCAACCT |
| 695 | (TA)_8_ | 59.8 | AGAAAAGCGAAAGCACAAGC | CTTAAATGCGCCACCAATTT |
| 700 | (TC)_9_ | 60.5 | TCCTCCTTTTAATCCCTCGC | GAATCCATCAACTGGGTGCT |
| 701 | (TTC)_7_ | 60.2 | TGGCTTGCTCTTTCTGTGTG | ATGGGACTCGCTTTGAGAGA |
| 703 | (GGC)_5_ | 60.0 | CTGAGACTAAGAGTGGCGGG | AAACCCACTGGACGTTTCTG |
| 707 | (CT)_10_ | 59.5 | CCCTCTTGACATCACAACCA | AGCTTTGAAGCCCTTGTTGA |
| 711 | (CT)_7_ | 62.4 | AGCGCTCATCCGCCATAG | GCGAAATCAAAGAGTCTCGG |
